# 2-Amino­pyridinium 1-phenyl­cyclo­propane-1-carboxyl­ate

**DOI:** 10.1107/S1600536810049093

**Published:** 2010-11-27

**Authors:** Guangwen He, Srinivasulu Aitipamula, Pui Shan Chow, Reginald B. H. Tan

**Affiliations:** a Institute of Chemical and Engineering Sciences, A*STAR (Agency for Science, Technology and Research), 1 Pesek Road, Jurong Island, Singapore 627833; b Department of Chemical & Biomolecular Engineering, National University of Singapore, 4 Engineering Drive 4, Singapore 117576

## Abstract

In the title salt, C_5_H_7_N_2_^+^·C_10_H_9_O_2_^−^, 2-amino­pyridine and 1-phenyl­cyclo­propane-1-carb­oxy­lic acid crystallize together, forming a 2-amino­pyridinium–carboxyl­ate supra­molecular heterosynthon involving two N—H⋯O hydrogen bonds, which in turn dimerizes to form a four-component supra­molecular unit also sustained by N—H⋯O hydrogen bonding. A C—H⋯π inter­action between a pyridine C—H group and the centroid of the phenyl ring of the anion further stabilizes the four-component supra­molecular unit. The overall crystal packing also features C—H⋯O inter­actions.

## Related literature

For structural studies of 2-amino­pyridine, see: Chao *et al.* (1975 ▶). For recent mol­ecular co-crystals and salts of 2-amino­pyridine, see: Sivaramkumar *et al.* (2010 ▶); Chitra *et al.* (2008 ▶); Quah *et al.* (2008 ▶); Xie (2007 ▶); Li *et al.* (2006 ▶, 2007 ▶); Yang & Qu (2006 ▶); Bis & Zaworotko (2005 ▶). For the use of 2-amino­pyridine in the synthesis of pharmaceuticals, see: O’Neil (2006 ▶). For our previous work on screening for molecular co-crystals and salts, see: He *et al.* (2009 ▶).
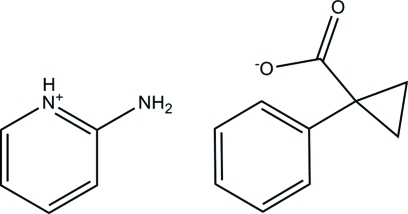


## Experimental

### 

#### Crystal data

C_5_H_7_N_2_^+^·C_10_H_9_O_2_^−^
*M* *_r_* = 256.30
Triclinic, 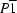

*a* = 8.6147 (17) Å
*b* = 9.0555 (18) Å
*c* = 9.2346 (18) Å
α = 75.56 (3)°
β = 87.72 (3)°
γ = 72.79 (3)°
*V* = 666.0 (2) Å^3^
*Z* = 2
Mo *K*α radiation
μ = 0.09 mm^−1^
*T* = 110 K
0.33 × 0.33 × 0.22 mm

#### Data collection

Rigaku Saturn CCD area-detector diffractometer
Absorption correction: multi-scan (Blessing, 1995 ▶) *T*_min_ = 0.972, *T*_max_ = 0.981
9557 measured reflections
3271 independent reflections
3103 reflections with *I* > 2σ(*I*)
*R*_int_ = 0.017

#### Refinement

*R*[*F* ^2^ > 2σ(*F* ^2^)] = 0.052
*wR*(*F* ^2^) = 0.145
*S* = 1.12
3271 reflections
184 parameters
H atoms treated by a mixture of independent and constrained refinement
Δρ_max_ = 0.31 e Å^−3^
Δρ_min_ = −0.24 e Å^−3^

### 

Data collection: *CrystalClear* (Rigaku, 2008 ▶); cell refinement: *CrystalClear*; data reduction: *CrystalClear*; program(s) used to solve structure: *SHELXS97* (Sheldrick, 2008 ▶); program(s) used to refine structure: *SHELXL97* (Sheldrick, 2008 ▶); molecular graphics: *X-SEED* (Barbour, 2001 ▶); software used to prepare material for publication: *SHELXTL* (Sheldrick, 2008 ▶) and *PLATON* (Spek, 2009 ▶).

## Supplementary Material

Crystal structure: contains datablocks global, I. DOI: 10.1107/S1600536810049093/ng5075sup1.cif

Structure factors: contains datablocks I. DOI: 10.1107/S1600536810049093/ng5075Isup2.hkl

Additional supplementary materials:  crystallographic information; 3D view; checkCIF report

## Figures and Tables

**Table 1:** Hydrogen-bond geometry (Å, °) *Cg*1 is the centroid of the C10–C15 ring.

*D*—H⋯*A*	*D*—H	H⋯*A*	*D*⋯*A*	*D*—H⋯*A*
N1—H1⋯O1	0.983 (19)	1.64 (2)	2.6255 (15)	176.2 (17)
N2—H6⋯O2^i^	0.913 (18)	1.938 (18)	2.8230 (16)	162.6 (16)
N2—H9⋯O2	0.949 (19)	1.844 (19)	2.7903 (15)	175.2 (16)
C11—H11⋯O1^ii^	0.95	2.53	3.479 (2)	178
C12—H12⋯O1^iii^	0.95	2.47	3.3212 (18)	149
C2—H2⋯*Cg*1^i^	0.95	2.62	3.5464 (15)	166

Symmetry codes: (i) 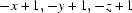; (ii) 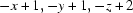; (iii) 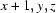.

**Fig. 1.**
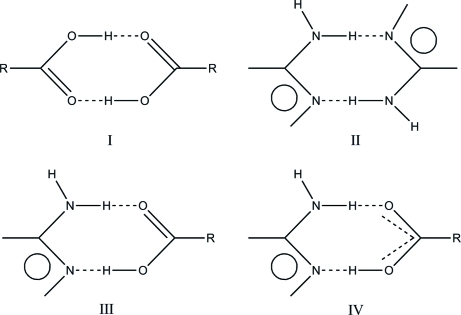
The molecular structures of 2-aminopyridinium ion and 1-phenylcyclopropane-1-carboxylate, with atom labels and 50% probability displacement ellipsoids for non-H atoms.

**Fig. 2.**
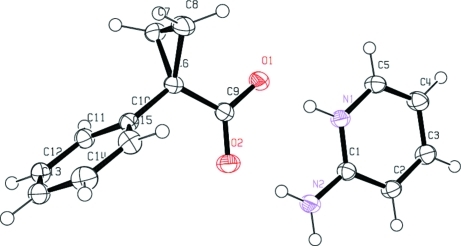
Supramolecular synthons: I. a carboxylic acid homosynthon; II. 2-aminopyridine homosynthon; III. 2-aminopyridine-carboxylic acid heterosynthon; IV. 2-aminopyridine-carboxylate heterosynthon.

**Fig. 3.**
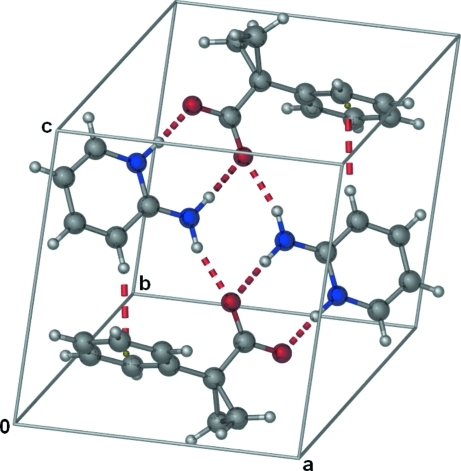
A four-component supramolecular unit that features heterosynthon IV and a C–H···π interaction in the crystal structure of the title salt.

**Fig. 4.**
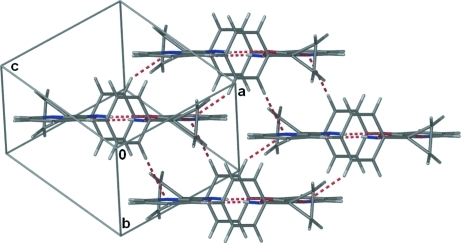
Part of the crystal structure of the title salt, showing the arrangement of the four-component supramolecular units which are stabilized by C–H···O interactions.

## supplementary crystallographic information

### Comment

2-Aminopyridine is one of the three positional isomers of aminopyridine and is widely used as an intermediate in the synthesis of pharmaceuticals (O'Neil, 2006). We have chosen 2-aminopyridine and 1-phenylcyclopropane-1-carboxylic acid for cocrystallization experiment as an extension work to our previous study on screening for molecular cocrystals and salts (He *et al.*, 2009).

The crystal structure of the title salt contains each one molecule of 2-aminopyridine and 1-phenylcyclopropane-1-carboxylic acid in the asymmetric unit (Fig. 2). Bis and Zaworotko revealed four types of two-point recognition possibilities in molecular complexes formed between 2-aminopyridine and carboxylic acids (Bis & Zaworotko, 2005). They have distinguished the type I–IV (Fig 1) synthons depending on whether the interacting complementary functional groups are the same or different. Type I involves formation of the carboxylic acid homosynthon; type II involves formation of the 2-aminopyridine homosynthon; type III and IV involve formation of 2-aminopyridine-carboxylic acid and 2-aminopyridinium-carboxylate supramolecular heterosynthons, respectively.

The title salt features type IV heterosynthon, where the 2-aminopyridinium ion forms a heterosynthon with the carboxylate group of the 1-phenylcyclopropane-1-carboxylate *via* two N–H···O (N···O = 2.6255 (15) and 2.7903 (15) Å) hydrogen bonds. Two such heterosynthons related by an inversion center dimerizes to form a four-component supramolecular unit sustained by N–H···O (N···O = 2.8230 (16) Å) hydrogen bonding (Fig. 3). The four-component supramolecular unit is further stabilized by a C–H···π interaction involving the 2-C–H of the pyridine ring and centroid of the phenyl ring of the carboxylate: C···*Cg1* (1 - *x*,1 - *y*,1 - *z*) = 3.5464 (15) Å, where *Cg1* denotes the centroid of the ring C10–C15 of 1-phenylcyclopropane-1-carboxylate (Fig. 4). Two prominent C–H···O interactions that involve the 11-C—H and 12-C—H of the phenyl ring of the 1-phenylcyclopropane-1-carboxylate and O1 of the same molecule at (-*x* + 1, -*y* + 1, -*z* + 2) and (*x* + 1, *y*, *z*), respectively, stabilize the overall crystal structure.

### Experimental

0.1883 g (2 mmol) of 2-aminopyridine (Acros Organic, 99+%)) and 0.3246 g (2 mmol) of 1-phenylcyclopropane-1-carboxylic acid (Sigma, 97%) and were dissolved into 3 ml of ethyl acetate (Fisher Scientific, HPLC). Solution was then filtered through a 0.22µm PTFE filter. Filtered solution was finally sealed with Parafilm^?^ and small holes were made to allow solvent to slowly evaporate. The block-shaped crystal (0.33 × 0.33 × 0.22 mm) suitable for single-crystal X-ray diffraction was collected after one day.

### Refinement

H atoms bonded to N and O atoms were located in a difference map and allowed to ride on their parent atoms in the refinement cycles. Other H atoms were positioned geometrically and refined using a riding model.

### Crystal data

**Table:**

C_5_H_7_N_2_^+^·C_10_H_9_O_2_^−^	*Z* = 2
*M**_r_* = 256.30	*F*(000) = 272
Triclinic, *P*1	*D*_x_ = 1.278 Mg m^−^^3^
Hall symbol: -P 1	Mo *K*α radiation, λ = 0.71073 Å
*a* = 8.6147 (17) Å	Cell parameters from 1840 reflections
*b* = 9.0555 (18) Å	θ = 2.3–30.9°
*c* = 9.2346 (18) Å	µ = 0.09 mm^−^^1^
α = 75.56 (3)°	*T* = 110 K
β = 87.72 (3)°	Block, colorless
γ = 72.79 (3)°	0.33 × 0.33 × 0.22 mm
*V* = 666.0 (2) Å^3^	

### Data collection

**Table:**

Rigaku Saturn CCD area-detector diffractometer	3271 independent reflections
Radiation source: fine-focus sealed tube	3103 reflections with *I* > 2σ(*I*)
graphite	*R*_int_ = 0.017
ω scans	θ_max_ = 28.3°, θ_min_ = 2.3°
Absorption correction: multi-scan (Blessing, 1995)	*h* = −11→11
*T*_min_ = 0.972, *T*_max_ = 0.981	*k* = −11→11
9557 measured reflections	*l* = −12→12

### Refinement

**Table:**

Refinement on *F*^2^	Primary atom site location: structure-invariant direct methods
Least-squares matrix: full	Secondary atom site location: difference Fourier map
*R*[*F*^2^ > 2σ(*F*^2^)] = 0.052	Hydrogen site location: inferred from neighbouring sites
*wR*(*F*^2^) = 0.145	H atoms treated by a mixture of independent and constrained refinement
*S* = 1.12	*w* = 1/[σ^2^(*F*_o_^2^) + (0.0812*P*)^2^ + 0.1565*P*] where *P* = (*F*_o_^2^ + 2*F*_c_^2^)/3
3271 reflections	(Δ/σ)_max_ < 0.001
184 parameters	Δρ_max_ = 0.31 e Å^−^^3^
0 restraints	Δρ_min_ = −0.24 e Å^−^^3^

### Special details

**Table:**

Geometry. All e.s.d.'s (except the e.s.d. in the dihedral angle between two l.s. planes) are estimated using the full covariance matrix. The cell e.s.d.'s are taken into account individually in the estimation of e.s.d.'s in distances, angles and torsion angles; correlations between e.s.d.'s in cell parameters are only used when they are defined by crystal symmetry. An approximate (isotropic) treatment of cell e.s.d.'s is used for estimating e.s.d.'s involving l.s. planes.
Refinement. Refinement of *F*^2^ against ALL reflections. The weighted *R*-factor *wR* and goodness of fit *S* are based on *F*^2^, conventional *R*-factors *R* are based on *F*, with *F* set to zero for negative *F*^2^. The threshold expression of *F*^2^ > σ(*F*^2^) is used only for calculating *R*-factors(gt) *etc*. and is not relevant to the choice of reflections for refinement. *R*-factors based on *F*^2^ are statistically about twice as large as those based on *F*, and *R*- factors based on ALL data will be even larger.

### Fractional atomic coordinates and isotropic or equivalent isotropic displacement parameters (Å^2^)

**Table:**

	*x*	*y*	*z*	*U*_iso_*/*U*_eq_	
O1	0.24208 (10)	0.61579 (11)	0.84154 (10)	0.0282 (2)	
N1	0.14380 (12)	0.42572 (12)	0.71820 (11)	0.0242 (2)	
O2	0.43518 (12)	0.64375 (12)	0.67744 (10)	0.0338 (2)	
N2	0.35609 (13)	0.42026 (15)	0.55735 (13)	0.0309 (3)	
C1	0.22273 (14)	0.37913 (14)	0.60017 (13)	0.0238 (2)	
C6	0.41282 (13)	0.76759 (14)	0.87938 (13)	0.0234 (2)	
C3	0.02113 (16)	0.25068 (15)	0.57653 (14)	0.0290 (3)	
H3	−0.0231	0.1918	0.5263	0.035*	
C9	0.36026 (13)	0.66849 (14)	0.79228 (13)	0.0235 (2)	
C11	0.71088 (14)	0.73844 (14)	0.88508 (13)	0.0246 (2)	
H11	0.7259	0.6408	0.9589	0.029*	
C10	0.55559 (14)	0.82560 (14)	0.82490 (12)	0.0230 (2)	
C12	0.84456 (14)	0.79240 (15)	0.83861 (13)	0.0268 (3)	
H12	0.9499	0.7318	0.8806	0.032*	
C2	0.15868 (15)	0.28868 (14)	0.52668 (13)	0.0257 (3)	
H2	0.2114	0.2548	0.4432	0.031*	
C5	0.00793 (15)	0.38539 (15)	0.76921 (13)	0.0269 (3)	
H5	−0.0431	0.4190	0.8534	0.032*	
C14	0.67030 (17)	1.02220 (15)	0.66824 (15)	0.0319 (3)	
H14	0.6560	1.1190	0.5934	0.038*	
C15	0.53697 (15)	0.96742 (15)	0.71553 (14)	0.0292 (3)	
H15	0.4320	1.0277	0.6725	0.035*	
C4	−0.05647 (16)	0.29794 (16)	0.70228 (14)	0.0304 (3)	
H4	−0.1509	0.2694	0.7390	0.036*	
C7	0.39043 (16)	0.72252 (17)	1.04712 (14)	0.0321 (3)	
H7A	0.3474	0.6310	1.0878	0.038*	
H7B	0.4712	0.7340	1.1135	0.038*	
C13	0.82364 (15)	0.93486 (15)	0.73082 (14)	0.0288 (3)	
H13	0.9145	0.9725	0.6999	0.035*	
C8	0.27730 (15)	0.87131 (17)	0.95326 (15)	0.0332 (3)	
H8A	0.2883	0.9746	0.9617	0.040*	
H8B	0.1645	0.8716	0.9359	0.040*	
H6	0.407 (2)	0.393 (2)	0.475 (2)	0.038 (4)*	
H9	0.389 (2)	0.492 (2)	0.600 (2)	0.041 (5)*	
H1	0.185 (2)	0.496 (2)	0.762 (2)	0.044 (5)*	

### Atomic displacement parameters (Å^2^)

**Table:**

	*U*^11^	*U*^22^	*U*^33^	*U*^12^	*U*^13^	*U*^23^
O1	0.0256 (4)	0.0351 (5)	0.0315 (5)	−0.0151 (3)	0.0083 (3)	−0.0157 (4)
N1	0.0263 (5)	0.0268 (5)	0.0226 (5)	−0.0103 (4)	0.0047 (4)	−0.0094 (4)
O2	0.0381 (5)	0.0433 (5)	0.0329 (5)	−0.0232 (4)	0.0150 (4)	−0.0210 (4)
N2	0.0292 (5)	0.0413 (6)	0.0326 (6)	−0.0188 (5)	0.0110 (4)	−0.0193 (5)
C1	0.0240 (5)	0.0242 (5)	0.0238 (5)	−0.0080 (4)	0.0027 (4)	−0.0063 (4)
C6	0.0212 (5)	0.0272 (6)	0.0249 (5)	−0.0082 (4)	0.0047 (4)	−0.0113 (4)
C3	0.0351 (6)	0.0295 (6)	0.0286 (6)	−0.0172 (5)	0.0054 (5)	−0.0097 (5)
C9	0.0213 (5)	0.0257 (5)	0.0256 (5)	−0.0078 (4)	0.0033 (4)	−0.0093 (4)
C11	0.0257 (6)	0.0256 (5)	0.0247 (5)	−0.0100 (4)	0.0033 (4)	−0.0078 (4)
C10	0.0238 (5)	0.0249 (5)	0.0244 (5)	−0.0094 (4)	0.0043 (4)	−0.0113 (4)
C12	0.0242 (5)	0.0316 (6)	0.0292 (6)	−0.0113 (4)	0.0030 (4)	−0.0123 (5)
C2	0.0302 (6)	0.0265 (6)	0.0234 (5)	−0.0111 (4)	0.0047 (4)	−0.0092 (4)
C5	0.0292 (6)	0.0286 (6)	0.0245 (5)	−0.0105 (5)	0.0073 (4)	−0.0082 (4)
C14	0.0403 (7)	0.0248 (6)	0.0318 (6)	−0.0137 (5)	0.0059 (5)	−0.0051 (5)
C15	0.0287 (6)	0.0260 (6)	0.0321 (6)	−0.0074 (5)	0.0009 (5)	−0.0065 (5)
C4	0.0323 (6)	0.0324 (6)	0.0314 (6)	−0.0169 (5)	0.0089 (5)	−0.0090 (5)
C7	0.0325 (6)	0.0468 (8)	0.0260 (6)	−0.0199 (6)	0.0090 (5)	−0.0167 (5)
C13	0.0326 (6)	0.0318 (6)	0.0308 (6)	−0.0182 (5)	0.0092 (5)	−0.0144 (5)
C8	0.0276 (6)	0.0372 (7)	0.0429 (7)	−0.0110 (5)	0.0115 (5)	−0.0242 (6)

### Geometric parameters (Å, °)

**Table:**

O1—C9	1.2702 (14)	C11—H11	0.9500
N1—C1	1.3524 (15)	C10—C15	1.3927 (17)
N1—C5	1.3597 (15)	C12—C13	1.3870 (18)
N1—H1	0.983 (19)	C12—H12	0.9500
O2—C9	1.2530 (15)	C2—H2	0.9500
N2—C1	1.3261 (15)	C5—C4	1.3595 (18)
N2—H6	0.913 (18)	C5—H5	0.9500
N2—H9	0.949 (19)	C14—C13	1.385 (2)
C1—C2	1.4178 (17)	C14—C15	1.3947 (17)
C6—C10	1.4977 (15)	C14—H14	0.9500
C6—C9	1.5122 (16)	C15—H15	0.9500
C6—C7	1.5201 (17)	C4—H4	0.9500
C6—C8	1.5224 (17)	C7—C8	1.491 (2)
C3—C2	1.3614 (16)	C7—H7A	0.9900
C3—C4	1.4125 (18)	C7—H7B	0.9900
C3—H3	0.9500	C13—H13	0.9500
C11—C10	1.3918 (17)	C8—H8A	0.9900
C11—C12	1.3922 (16)	C8—H8B	0.9900
			
C1—N1—C5	121.81 (10)	C3—C2—C1	119.58 (11)
C1—N1—H1	117.1 (11)	C3—C2—H2	120.2
C5—N1—H1	121.0 (11)	C1—C2—H2	120.2
C1—N2—H6	118.9 (11)	C4—C5—N1	121.41 (11)
C1—N2—H9	120.8 (11)	C4—C5—H5	119.3
H6—N2—H9	119.5 (15)	N1—C5—H5	119.3
N2—C1—N1	118.89 (11)	C13—C14—C15	119.75 (12)
N2—C1—C2	122.65 (11)	C13—C14—H14	120.1
N1—C1—C2	118.46 (11)	C15—C14—H14	120.1
C10—C6—C9	117.44 (9)	C10—C15—C14	120.94 (12)
C10—C6—C7	118.19 (10)	C10—C15—H15	119.5
C9—C6—C7	115.04 (10)	C14—C15—H15	119.5
C10—C6—C8	118.76 (10)	C5—C4—C3	118.07 (11)
C9—C6—C8	115.60 (10)	C5—C4—H4	121.0
C7—C6—C8	58.71 (9)	C3—C4—H4	121.0
C2—C3—C4	120.64 (11)	C8—C7—C6	60.72 (9)
C2—C3—H3	119.7	C8—C7—H7A	117.7
C4—C3—H3	119.7	C6—C7—H7A	117.7
O2—C9—O1	124.47 (11)	C8—C7—H7B	117.7
O2—C9—C6	118.39 (10)	C6—C7—H7B	117.7
O1—C9—C6	117.13 (10)	H7A—C7—H7B	114.8
C10—C11—C12	120.92 (11)	C14—C13—C12	120.03 (11)
C10—C11—H11	119.5	C14—C13—H13	120.0
C12—C11—H11	119.5	C12—C13—H13	120.0
C11—C10—C15	118.48 (11)	C7—C8—C6	60.57 (8)
C11—C10—C6	120.06 (10)	C7—C8—H8A	117.7
C15—C10—C6	121.46 (11)	C6—C8—H8A	117.7
C13—C12—C11	119.87 (11)	C7—C8—H8B	117.7
C13—C12—H12	120.1	C6—C8—H8B	117.7
C11—C12—H12	120.1	H8A—C8—H8B	114.8

### Hydrogen-bond geometry (Å, °)

**Table:**

Cg1 is the centroid of the C10–C15 ring.

**Table:**

*D*—H···*A*	*D*—H	H···*A*	*D*···*A*	*D*—H···*A*
N1—H1···O1	0.983 (19)	1.64 (2)	2.6255 (15)	176.2 (17)
N2—H6···O2^i^	0.913 (18)	1.938 (18)	2.8230 (16)	162.6 (16)
N2—H9···O2	0.949 (19)	1.844 (19)	2.7903 (15)	175.2 (16)
C11—H11···O1^ii^	0.95	2.53	3.479 (2)	178
C12—H12···O1^iii^	0.95	2.47	3.3212 (18)	149
C2—H2···Cg1^i^	0.95	2.62	3.5464 (15)	166

Symmetry codes: (i) −*x*+1, −*y*+1, −*z*+1; (ii) −*x*+1, −*y*+1, −*z*+2; (iii) *x*+1, *y*, *z*.
